# Seizure Outcome After Intraoperative Electrocorticography-Tailored Epilepsy Surgery: A Systematic Review and Meta-Analysis

**DOI:** 10.1212/WNL.0000000000209430

**Published:** 2024-05-20

**Authors:** Jiaojiao Guo, Ziyi Wang, Maryse A. van 't Klooster, Sandra M. Van Der Salm, Frans S. Leijten, Kees P. Braun, Maeike Zijlmans

**Affiliations:** From the Department of Neurology and Neurosurgery (J.G., Z.W., M.A.K., S.M.V.D.S., F.S.L., K.P.B., M.Z.), University Medical Center Utrecht Brain Center, University Medical Center Utrecht, Part of ERN EpiCARE; and Stichting Epilepsie Instellingen Nederland (SEIN) (M.Z.), the Netherlands.

## Abstract

**Background and Objectives:**

Tailoring epilepsy surgery using intraoperative electrocorticography (ioECoG) has been debated, and modest number of epilepsy surgery centers apply this diagnostic method. We assessed the current evidence to use ioECoG-tailored epilepsy surgery for improving postsurgical outcome.

**Methods:**

PubMed and Embase were searched for original studies reporting on ≥10 cases who underwent ioECoG-tailored surgery for epilepsy, with a follow-up of at least 6 months. We used a random-effects model to calculate the overall rate of patients achieving favorable seizure outcome (FSO), defined as Engel class I, ILAE class 1, or seizure-free status. Meta-regression was used to investigate potential sources of heterogeneity. We calculated the odds ratio (OR) for estimating variables on FSO:ioECoG vs non-ioECoG-tailored surgery (if included studies contained patients with non-ioECoG-tailored surgery), ioECoG-tailored epilepsy surgery in children vs adults, temporal (TL) vs extratemporal lobe (eTL), MRI-positive vs MRI-negative, and complete vs incomplete resection of tissue that generated interictal epileptiform discharges (IEDs). A Bayesian network meta-analysis was conducted for underlying pathologies. We assessed the evidence certainty using the Grading of Recommendations, Assessment, Development, and Evaluation (GRADE).

**Results:**

Eighty-three studies (82 observational studies, 1 trial) comprising 3,631 patients with ioECoG-tailored surgery were included. The overall pooled rate of patients who attained FSO after ioECoG-tailored surgery was 74% (95% CI 71–77) with significant heterogeneity, which was predominantly attributed to pathologies and seizure outcome classifications. Twenty-two studies contained non–ioECoG-tailored surgeries. IoECoG-tailored surgeries reached a higher rate of FSO than non–ioECoG-tailored surgeries (OR 2.10 [95% CI 1.37–3.24]; *p* < 0.01; very low certainty). Complete resection of tissue that displayed IEDs in ioECoG predicted FSO better compared with incomplete resection (OR 3.04 [1.76–5.25]; *p* < 0.01; low certainty). We found insignificant difference in FSO after ioECoG-tailored surgery in children vs adults, TL vs eTL, or MRI-positive vs MRI-negative. The network meta-analysis showed that the odds of FSO was lower for malformations of cortical development than for tumors (OR 0.47 95% credible interval 0.25–0.87).

**Discussion:**

Although limited by low-quality evidence, our meta-analysis shows a relatively good surgical outcome (74% FSO) after epilepsy surgery with ioECoG, especially in tumors, with better outcome for ioECoG-tailored surgeries in studies describing both and better outcome after complete removal of IED areas.

## Introduction

Surgery is an effective treatment for people with drug-resistant epilepsy.^1^ Seizure freedom is achieved in 60%–76% at 1 year after surgery.^2-5^ Success depends on epileptogenic tissue being precisely delineated and removed. Intraoperative electrocorticography (ioECoG) offers neurosurgeons instantaneous readouts of pathologic neuronal activity.^6^ It may help to delineate the epileptogenic brain region, especially in focal and superficial lesions, map out functional areas, and identify residual epileptiform abnormalities after the initial surgical process.^7,8^

IoECoG has been practiced in epilepsy surgery centers around the world for more than half a century.^9^ Resections are tailored by identifying interictal epileptiform discharges (IEDs) and rhythmic patterns pre resection and post resection.^10^ IoECoG tailoring may identify surrounding epileptic tissue alongside a lesionectomy and adapt the surgical plan. Some centers use it for several scenarios (e.g., verifying neocortical involvement next to a primary mesiotemporal focus and assessing resection extent in neocortical lesions) next to diagnostic long-term invasive EEG for seizure-onset zone (SOZ) localization.^11^ However, IEDs on ioECoG may be discordant with the SOZ.^12-14^ IEDs are prone to appear due to surgical irritation after resection.^15,16^ Due to its interictal character, shorter duration, and limited ability to capture activity from deeper brain structures, the use of ioECoG decreased with the introduction of long-term invasive stereo-EEG, especially in Western countries. Recent technical developments toward easy high-density electrode-grid manufacturing and the rise in newly identified interictal biomarkers may be reasons to revive popularity. Accessing frequencies above 80 Hz and advanced signal analysis methods, such as phase amplitude coupling, entropy, and connectivity measures, yield potential interictal biomarkers.^17-19^ In case of clear-defined neocortical lesions, using ioECoG tailoring may prevent complications from diagnostic long-term invasive EEG.^8,20,21^

Reported favorable seizure outcome (FSO) after ioECoG-guided surgery ranges from 31% to 93%.^22,23^ A recent patient-level meta-analyses (individual participant data meta-analysis [IPDMA]) of 18 studies showed more FSO after surgeries with than without ioECoG of and a high benefit in focal cortical dysplasia (FCD).^24^ Due to the limited number of studies, they did not explore clinical variables affecting postsurgical FSO rate in patients tailored by ioECoG. We conducted systematic review and meta-analysis including a wider range of articles reporting on ioECoG-tailored surgery to (1) estimate the overall FSO rate of ioECoG-tailored surgery; (2) compare the success rate of FSO between surgeries with and without ioECoG; and (3) analyze potential determinants of seizure outcome, including completeness of resection of tissue showing biomarkers, age at surgery, MRI characteristics, surgery location, and pathology.

## Methods

### Literature Search Strategy

**Figure 1:**
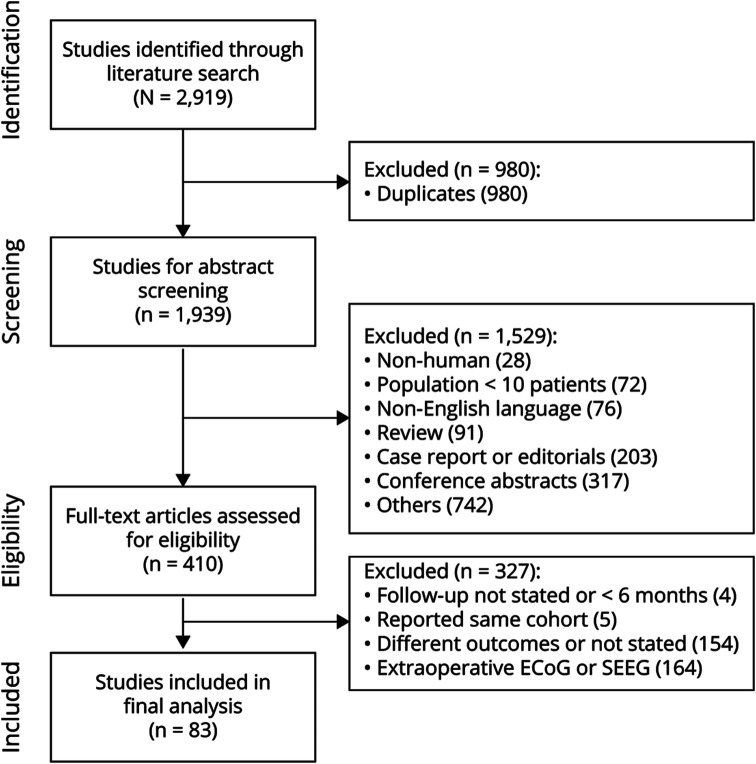
PRISMA Flowchart of the Literature Search and Study Selection

The literature search and study design was planned and conducted in accordance with the Preferred Reporting Items for Systematic Reviews and Meta-Analyses (PRISMA) guidelines. Two databases, PubMed and EMBASE, were used to perform a literature search on March 7, 2022, without an initial date limitation, which was updated on October 28, 2022. The search keywords *epilepsy*, *electrocorticography*, and *surgery* and their synonyms and variant terms were combined using the Boolean AND between them (details in eMethods). The article selection process is summarized in a PRISMA flowchart (Figure 1). Reference lists of included studies were manually searched for potentially missed article (snowballing).

### Selection of Studies

We included randomized controlled trials (RCTs) and observational studies with prospective or retrospective design with a population size of ≥10 patients who underwent ioECoG-tailored surgery with at least 6 months of postsurgical follow-up. Eligible studies containing control cohorts of non–ioECoG-tailored surgeries were used in the subgroup analysis. Given the absence of a standardized control group, we used *non–ioECoG-tailored surgery* as the name of comparator. Non–ioECoG-tailored does not refer to extraoperative ECoG.

The following reports were excluded: reviews, unpublished data, notes, correspondences, editorials, letters, case reports, nonhuman, and articles with full-text unavailability. Other exclusion criteria consisted of studies on overlapping research populations from the same center, publications in non-English language, studies in which the distinction between people who underwent ioECoG and those who underwent extraoperative ECoG could not be made, and those in which ioECoG was solely intended for functional mapping.

### Data Collection

One investigator conducted the literature search and initial screening (J.G.). Duplicate records were removed, and remaining articles were screened by titles and abstracts based on eligibility and exclusion criteria. Full texts of selected studies were then reviewed by 2 reviewers (J.G. and Z.W.), and any issues of disagreement involving eligibility was resolved by consensus.

Data from eligible studies were collected into predefined data extraction Excel spreadsheets. Information on first author, year of publication, country, study design, population, sex, number of patients, type of anesthetics and electrodes, used biomarkers for tailoring, duration of ioECoG recording, seizure outcome classification, and duration of follow-up was assembled from each study (eTable 1). Per-patient data of non-ioECoG surgeries, age at surgery, preoperative MRI characteristics (MRI-positive or MRI-negative), epilepsy surgery location (temporal lobe [TL] or extratemporal lobe [eTL]), underlying pathology, and complete or incomplete resection of tissue displaying IEDs in ioECoG were extracted. Inconsistencies were resolved through discussion or, if necessary, by consulting a third researcher (M.K.).

### Outcome Measure

We classified FSO based on the classification used by individual studies (i.e., Engel class 1 [incl. 1 A–D], ILAE class 1, or seizure-free status [SFS], not otherwise specified). The last available follow-up was collected for analysis in case of multiple time points.

### Quality Assessment of Included Studies

We evaluated risks of bias per included observational study using the Newcastle-Ottawa Scale (NOS)^25^ in the domains: selection of cohort (0–4 stars), comparability between cohorts (0–2 stars), and outcomes (0–3 stars). The maximum was 9 stars, categorized as low (0–3), moderate (4–6), or high quality (7–9). Due to the NOS, its inapplicability of some parameters for studies without control groups, these studies were scored with a maximum of 5 stars, categorized as low (0–2), moderate (3), or high quality (4–5).^26^ The quality of RCTs was assessed using the Cochrane risk-of-bias tool.^27^

### Assessment of Evidence

Grading of Recommendations, Assessment, Development, and Evaluation (GRADE)^28^ assessed the quality of evidence rather than evaluating individual studies (eTable 2). We assessed the strength of evidence for all the dichotomous meta-analyses.

### Statistical Analysis

#### Meta-Analysis

Data were synthesized and analyzed to summarize the pooled FSO rate after ioECoG-tailored surgeries across the included studies, which was calculated implementing random-effects models with the DerSimonian-Laird method and Freeman-Tukey Double arcsine transformation. We presented it as overall FSO rate with a 95% CI. Subgroup analysis and univariate meta-regression analysis with a mixed-effects model were used to reveal probable sources of variability. We calculated R^2^ to assess the amount of heterogeneity variance that is accounted for by each independent variable. A higher value indicates the variable was able to explain a larger portion of the variability.

The odds ratio (OR) served as the effect size in meta-analysis to compare FSO of ioECoG vs non-ioECoG-tailored surgery, children vs adults, TL vs eTL, MRI-positive vs MRI-negative, and complete vs incomplete resection of tissue underlying ioECoG IEDs. In the abovementioned 5 meta-analyses, all patients except for the first subgroup originated from ioECoG-tailored surgery cohorts.

We implemented *I*^2^ and Q statistics to scrutinize heterogeneity. In dichotomous meta-analysis, when heterogeneity was not significant (I^2^ < 50% or *p* > 0.05), the fixed-effect model was used. If there was significant heterogeneity, the random-effects model was used for pooled estimate and 95% CI. The funnel plot and Egger test were applied to judge small study effects and publication bias.

#### Network Meta-Analysis (NMA) for Pathology

We categorized underlying pathology into 5 groups: tumor, malformations of cortical development (MCD), mesiotemporal sclerosis (MTS), dual pathology, and *others* (e.g., infarction, gliosis). Due to small sample sizes, normal pathology was included in *others*. The NMA was performed in a Bayesian framework to estimate OR with 95% credible interval (CrI). We used a random-effects model to evaluate the direct, indirect, and the mixed direct and indirect evidence between different pathologies. Direct evidence referred to comparing 2 of the 5 pathology groups within a single study. Indirect evidence compared FSO of 2 pathology groups (e.g., MCD, MTS) indirectly through a common comparator (e.g., tumor), where the pathologies were not directly compared in an individual study. Mixed direct and indirect evidence represented a combination of direct and indirect evidence. Consistency of the NMA was tested between direct and indirect evidence using node-splitting analysis.

The R studio (v.4.2.1) was used for all analyses. The meta package was used for meta-analysis. The metafor package was conducted for meta-regression. The NMA was generated using gemtc package. A *p* value of <0.05 was defined as statistical significance.

### Standard Protocol Approvals, Registrations, and Patient Consents

This study was registered with PROSPERO (registration number: CRD42022343123). No ethical standards committee approval and patient consent were required due to the nature of systematic review and meta-analysis.

### Data Availability

Relative data and code are available to qualified researchers on request to the corresponding author.

## Results

### Study Inclusion

The literature search strategy yielded 2,919 original studies, from which 410 articles were obtained and reviewed in full text for eligibility (Figure 1). We excluded 327 studies mostly because they did not distinguish between patients with extraoperative ECoG and ioECoG (n = 164). Other reasons for exclusion were not reporting—or using different criteria for—seizure freedom (n = 154), studies conducted on the same population (n = 5), and studies with a follow-up duration of less than 6 months or not reported (n = 4). The pertinent data were taken from 83 studies (publication dates: 1990–2022) left after being critically evaluated.

### Study Characteristics

All, except for 1 RCT,^29^ were retrospective observational studies comprising 7 case-control studies and 75 cohort studies. Of the 83 studies, 61 described cohorts of 2,769 patients in total undergoing ioECoG-tailored surgeries only and 22 included 862 ioECoG-tailored and 596 non–ioECoG-tailored surgeries. Reasons for (not) using ioECoG are listed in eTable 3, and 16 studies did not provide rationales. Seventy-two (86.8%) studies reported a variety of presurgical investigations, including scalp EEG, MRI, PET, SPECT, or magnetoencephalography. Seven studies mentioned using extraoperative invasive EEG in several patients. The recording of ioECoG was covered in detail by 32 studies (38.6%), while the other studies simply mentioned ioECoG as a method for tailored resection. The duration of ioECoG recordings ranged from 1 to 45 minutes. Forty-one (49.4%) studies provided information on the administration of anesthesia. Anesthesia was induced or maintained with propofol alone (11 studies) or in combination with other anesthetics (17 studies), with sevoflurane (alone = 4; combined = 14) or fentanyl (alone = 2; combined = 15), but the anesthetic protocol varied across studies and within studies. Standard ioECoG electrodes with 10-mm spacing between contacts were most prevalent; one study used high-density electrodes as well. Ictal or interictal epileptiform discharges were reported as indicators to delineate the resection in all studies; one study was an RCT comparing HFO-guided (38 patients) with spike-guided (38 patients) ioECoG tailoring.^29^ Sixty-eight studies reported seizure outcome based on Engel classifications; 56 studies distinguished Engel class I from II–IV, 5 studies Engel 1A from 1B–IV, and 7 studies Engel 1A and B from 1C–IV. Six studies reported ILAE scores, distinguishing ILAE class 1 from ILAE 2–6. Nine studies reported seizure freedom without further classification. In most studies (87%), postsurgical follow-up was ≥12 months (≥12 months = 49 studies; ≥48 months = 20 studies; and ≥60 months = 3 studies; Table 1).

**Table 1:** Study-Level Subgroup Pooled Rate Meta-Analyses and Univariate Meta-Regression of the Effect of Variables on Favorable Seizure Outcome After ioECoG-Tailored Epilepsy Surgery

Variable	Number of studies, n	Total no. of FSO, n	Total no. of patients, n	FSO percentage (95% CI), %	Heterogeneity		Univariate meta-regression	
					I^2^	*p* Value	*p* (test of variables)	R^2^ (variability accounted for)
Age at surgery (y)							0.47	0.0%
≤18 (children)	23	766	1,065	76 (70–82)	78%	<0.01		
>18 (adults)	11	260	341	78 (68–86)	71%	<0.01		
Mixed children and adults^a^	49	1,568	2,225	73 (68–77)	75%	<0.01		
Surgery location							0.08	2.3%
TL	30	889	1,263	75 (70–80)	72%	<0.01		
eTL	3	48	89	54 (43–64)	0%	0.55		
Mixed locations^a^	50	1,657	2,279	75 (70–79)	79%	<0.01		
Pathology							<0.05^b^	22.4%
Tumor	13	366	431	86 (83–90)	50%	0.02		
MCD	8	190	274	72 (62–81)	67%	0.03		
MTS	4	119	157	77 (69–83)	21%	0.28		
CMs	6	184	222	83 (78–88)	0%	0.80		
Mixed pathologies^c^	52	1,735	2,547	70 (66–74)	77%	<0.01		
MRI findings							0.36	3.2%
Positive	29	806	1,069	77 (72–81)	63%	<0.01		
Negative	3	139	231	62 (51–73)	56%	0.10		
Mixed or not reported^a^	51	1,649	2,331	74 (69–78)	80%	<0.01		
IoECoG change original surgery plan or not							0.45	0.1%
Reported	5	260	331	79 (74–83)	0%	0.74		
Not reported	78	2,334	3,300	74 (71–77)	77%	<0.01		
Completed resection (based on IEDs on ioECoG)							0.26	2.3%
Reported^a^	23	1,045	1,530	71 (65–76)	83%	<0.01		
Not reported	60	1,549	2,101	76 (72–79)	7%	<0.01		
Postsurgical seizure outcome classification							<0.05^b^	14.3%
Engel class 1 (A–D)	68	2,143	2,911	76 (72–79)	72%	<0.01		
ILAE classification	6	148	277	56 (47–66)	54%	0.05		
Seizure-free (no further specified)	9	303	443	74 (63–84)	84%	0.01		
Duration of postsurgical follow-up							0.19	8.6%
≥6 mo	11	275	340	80 (72–88)	63%	0.03		
≥1 y	49	1,476	2,169	72 (67–76)	78%	<0.01		
≥2 y	20	777	1,041	76 (70–81)	69%	<0.01		
≥5 y	3	66	81	84 (70–94)	50%	0.14		

Abbreviations: CMs = cavernous malformations; eTL = extratemporal lobe; FSO = favorable seizure outcome; ILAE = International League Against Epilepsy; ioECoG = intraoperative electrocorticography; MCD = malformations of cortical development (including focal cortical dysplasia and tuberous sclerosis); MTS = mesial temporal sclerosis; R^2^ = amount of variance accounted for; TL = temporal lobe.

a From these groups, studies that provided available data were selected for subsequent subgroup meta-analysis.

b *p* < 0.05.

c Studies included in the network meta-analysis from this group.

### Meta-Analyses

#### Meta-Analysis of the Pooled FSO Rate in ioECoG-Tailored Surgery

Data were pooled from all 83 studies with 3,631 patients who underwent ioECoG-tailored surgery. The overall pooled rate of FSO was 74% (95% CI 71%–77%) with statistically significant heterogeneity between studies (*I*^2^ = 75%, *p* < 0.01; eFigure 1). Univariate meta-regression disclosed that pathology and seizure outcome classifications were found to account for significant heterogeneity variance (R^2^ = 22.4% and 14.3%; both *p* < 0.05) (Table 1).

#### Meta-Analysis of Dichotomous Variables

##### ioECoG vs Non-ioECoG Tailored Surgery

**Figure 2:**
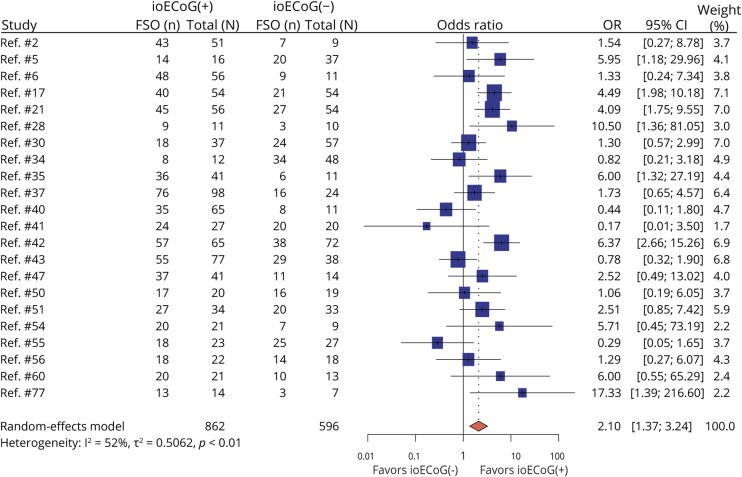
Forest Plot for Meta-Analysis of Favorable Seizure Outcome (FSO) Comparing Surgery With ioECoG Tailoring (ioECoG+) With That Without ioECoG Tailoring (ioECoG-) Pooled results (red diamond) show a higher odds to attain FSO based on ioECoG. FSO = favorable seizure outcome; ioECoG = intraoperative electrocorticography; n = the number of people attaining favorable seizure outcome; N = the total number of people; OR = odds ratio.

Twenty-two studies (eTable 3) compared rates of FSO in 862 patients with ioECoG-tailored surgery with those in 596 patients who underwent surgery without ioECoG. IoECoG tailoring reached a higher FSO rate than non-ioECoG (OR = 2.10 [95% CI 1.37–3.24]); Figure 2]).

##### Age at Surgery (Adults vs Children)

**Figure 3:**
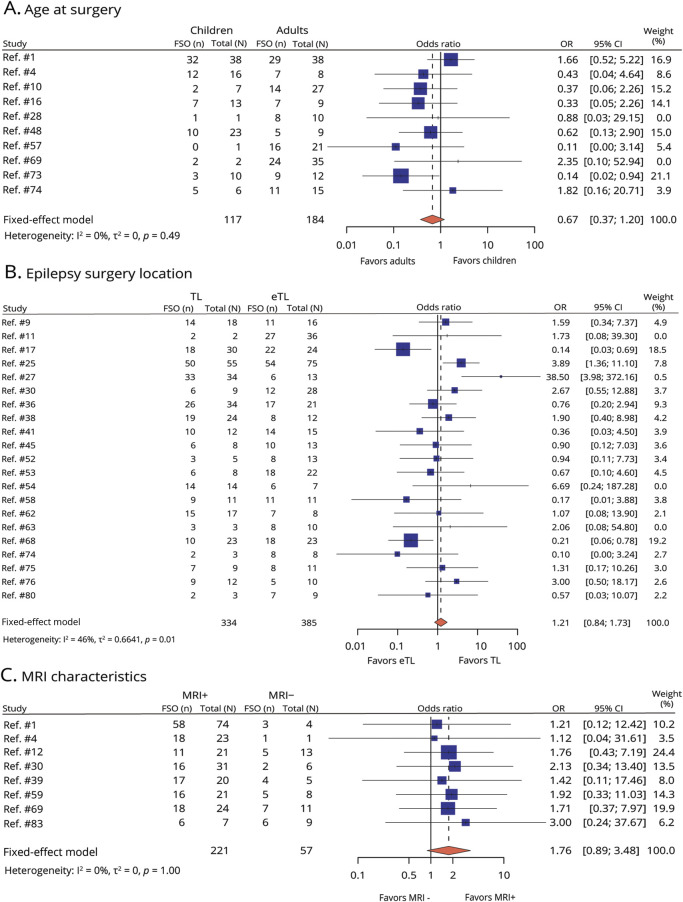
Forest Plot for Meta-Analysis of Favorable Seizure Outcome (FSO) Comparing (A) Children With Adults, (B) TL With eTL, and (C) MRI-Positive With MRI-Negative Results show no statistical difference between children and adults, TL and eTL, and a nonsignificant trend of the chance of FSO being smaller in people with MRI-negative epilepsy compared with those with MRI-positive epilepsy. Each square (blue) represents an independent study, and diamond (red) shows the pooled data with a 95% CI. eTL = extratemporal lobe; FSO = favorable seizure outcome; n = the number of people attaining favorable seizure outcome; N = the total number of people; OR = odds ratio; TL = temporal lobe.

The chance of FSO after ioECoG-guided surgery in 23 children cohorts was 76% (95% CI 70%–82%) and 78% in 11 adult cohorts (95% CI 68%–86%) (study-level analysis; Table 1). From the remaining 49 studies comprising mixed-age cohorts, we extracted relevant data from 10 studies (encompassing 117 children and 184 adults) and subsequently performed a dichotomous meta-analysis. The result showed that children and adults had a similar chance of attaining FSO (OR = 0.67 [95% CI 0.37–1.20; Figure 3A]).

##### Epilepsy Surgery Location (TL vs eTL)

In 30 studies on TL surgery cohorts, 75% (95% CI 70%–80%) of individuals achieved FSO after ioECoG-guided TL surgery. Three studies on eTL cohorts (include 2 with frontal lobe and 1 with various extratemporal regions) demonstrated a pooled FSO rate of 54% (95% CI 43%–64%) (Table 1). Of the remaining 50 studies, a total of 21 studies reported FSO for both TL (n = 334) and eTL (n = 385). FSO after ioECoG-tailored surgery was not influenced by surgery location (OR = 1.21 [95% CI 0.84–1.73; Figure 3B]).

##### MRI Characteristics (MRI-Positive vs MRI-Negative)

By pooled rate meta-analysis, we analyzed 29 studies that included only MRI-positive cohorts and obtained an FSO of 77% (95% CI 72%–81%); meanwhile, for 3 studies including only MRI-negative cohort, we found an FSO rate of 62% (95% CI 51%–73%) (Table 1). Eight of 51 studies with mixed MRI-positive and MRI-negative cohorts contained accessible data and were included in the dichotomous meta-analysis. MRI characteristics were not significantly associated with FSO (OR = 1.76 [95% CI 0.89–3.48; Figure 3C]), with 221 patients in the MRI-positive group and 57 patients in the MRI-negative group.

##### Resection of IED-Generating Tissue (Complete vs Incomplete)

**Figure 4:**
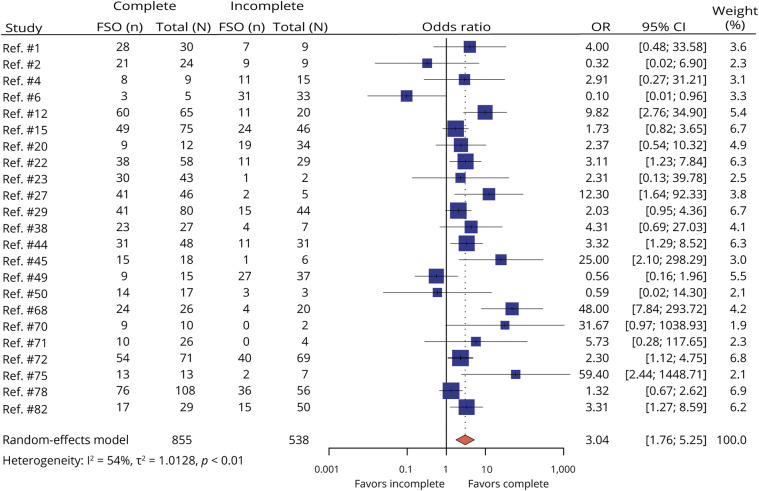
Forest Plot for Meta-Analysis of FSO Comparing Complete With Incomplete Resection of IEDs for Epileptogenic Tissue in the ioECoG Each square represents an independent study and diamonds show the pooled data with a 95% CI. FSO = favorable seizure outcome; n = the number of people attaining favorable seizure outcome; N = the total number of people; OR = odds ratio.

There were 23 studies that evaluated the effects of complete and incomplete removal of tissue that generates ioECoG biomarkers on FSO. Complete resection of IED-generating tissue (n = 855) increased the probability for FSO compared with incomplete resection of IEDs (n = 538) (OR = 3.04, 95% CI 1.76–5.25) (Figure 4).

##### Network Meta-Analysis for Pathologies

**Figure 5:**
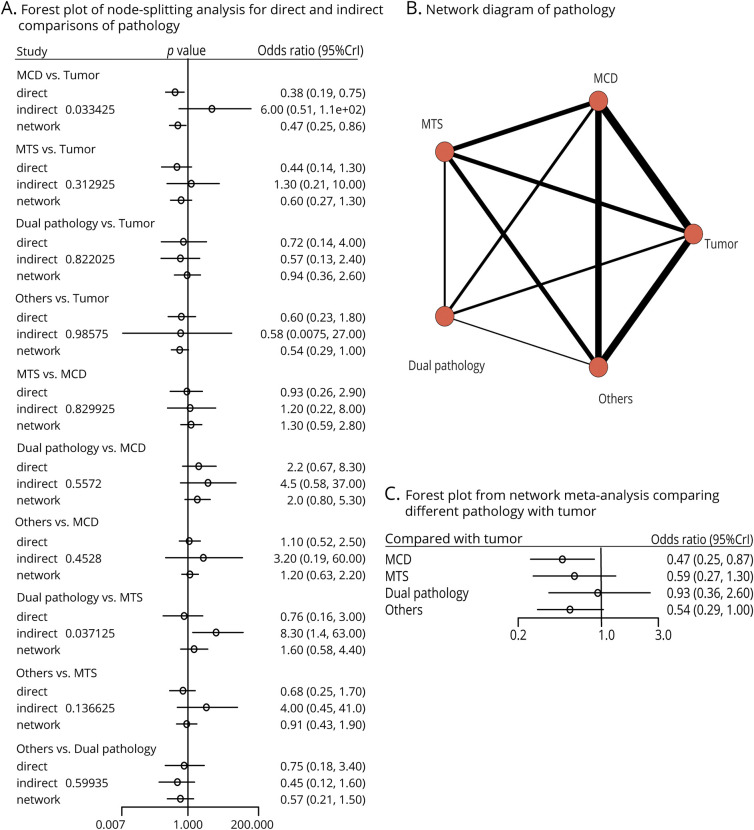
Results of the Network Meta-Analysis of the Underlying Pathologies Performed on 26 Studies (A) Forest plot of node-splitting analyses for direct and indirect comparisons of the 5 categories of underlying pathologies was presented. (B) A network diagram of the 5 pathology categories. Each red circle represents a pathology category. Each line is an edge, and its thickness corresponds to the number of studies that included the respective direct estimate. (C) Forest plot of the network meta-analysis for FSO for the different underlying pathology categories, comparing FSO in malformations of cortical development (MCD), mesiotemporal sclerosis (MTS), dual pathology, and others with FSO in tumors. CrI = credible interval; FSO = favorable seizure outcome.

FSO for ≥2 underlying pathology groups was described in 26 studies. Node-splitting analyses demonstrated the consistency between all direct and indirect comparisons except for MCD vs tumor and dual pathology vs MTS (Figure 5A; *p* < 0.05). A connected network diagram of pathologies implies that comparisons between different pathologies in network were either directly or indirectly connected to one another (Figure 5B). Compared with tumors, the chance of FSO was weaker for MCD (OR = 0.47 [95%CrI 0.25–0.87]) following ioECoG-tailored surgery, as demonstrated in the forest plot of NMA (Figure 5C). Instead, ioECoG-tailored surgery did not show significant inferiority in MTS (OR = 0.59 [95%CrI 0.27–1.3]), dual pathology (OR = 0.93 [95%CrI 0.36–2.6]), and others (OR = 0.54 [95%CrI 0.29–1.0]).

#### Quality of Evidence Assessment and Publication Bias

The RCT was of low risk according to the Cochrane risk of bias tool. For the quality of observational studies with the non-ioECoG group, the mean star was 6.09; for those studies without, the mean star was 3.48, indicating the study quality in between moderate and high based on NOS. The funnel plot and Egger test showed no evidence of publication bias (eFigure 2–7, eTable 4).

The certainty of evidence grading (GRADE) started at low because the meta-analyses consisted of observational studies. It was further downgraded to very low regarding the effectiveness of ioECoG, primarily because of a serious risk of bias (eTable 5). This suggests that we have limited confidence in the effect estimates. There was no justification for upgrading or downgrading the evidence level for age at surgery, epilepsy location, MRI characteristics, and (in)complete resection of IEDs (eTable 6–9).

## Discussion

We performed a quantitative meta-analysis on 83 studies with 3,631 patients and found that (1) 74% (95% CI 71%–77%) of patients with epilepsy achieved seizure freedom after ioECoG-tailored surgery; (2) ioECoG-tailored epilepsy surgery was more likely to achieve FSO than non-ioECoG surgery (OR = 2.1, 1.37–3.24) in studies that included both; and (3) complete resection of IED-generating tissue was associated with FSO (OR = 3.04, 1.76–5.25). These findings suggest that ioECoG may be able to help improve epilepsy surgery outcome. Furthermore, the FSO rate in people with tumors was higher than with those with MCDs.

The 74% FSO rate for ioECoG-tailored epilepsy surgery should be interpreted and generalized with caution, and it cannot serve as evidence for the value of ioECoG compared with diagnostic long-term invasive EEG. In several centers, ioECoG is available as a complementary method. Most studies included people with TLE, MRI lesions, or tumor-related epilepsy and thus *good candidates* for surgery.^11^ Patients with deep-seated foci, which may have unfavorable outcome for surgery—technical reasons, are unsuitable for (io)ECoG. A quarter of the included studies were from middle-income or low-income countries, for which the high costs of extraoperative ECoG might have led to opt for ioECoG. In these countries, only people with relative high incomes may afford ioECoG, limiting widespread use.

We encountered substantial heterogeneity that originated from different classifications of seizure outcome and distinct pathologies. We hypothesized that the main sources of heterogeneity were study design and quality, varying lengths of follow-up, and clinically relevant variables. By subgroup analysis and univariate meta-regression for clinical variables, we investigated 8 predefined variables and found 2 of them that statistically explained such significant heterogeneity, namely classification of postsurgical seizure outcome and diverse pathologies across different studies. The classifications of favorable postsurgical seizure outcome consisted of Engel class 1A–D, ILAE class 1, and SFS. Among them, ILAE class 1 was a more stringent criterion—being completely seizure-free without auras—compared with the other 2.^30^ This may explain why studies that used ILAE class 1 yielded the lowest FSO rate of 56%. In a meta-analysis by Widjaja et al., the FSO rate of tumor-related epilepsy surgery (79.8%) was higher than that for MCD-related epilepsy (57.1%).^31^ We found a similar distribution of FSO between patients with tumor and MCD (86% vs 72%) who underwent ioECoG-tailored surgery.

Our subanalysis demonstrated superior FSO rates of ioECoG-tailored surgery compared with non-ioECoG surgery at study level, which was not found by Goel et al.^24^ albeit a similar trend was observed (risk ratios = 1.09, 0.96–1.23). This discrepancy resulted from the inclusion of 4 recent publications. Selection bias may occur because patients in ioECoG and non-ioECoG tailored surgery groups were not randomly assigned. The decision to use ioECoG is usually made by neurologists. The epilepsies of patients undergoing non-ioECoG surgery are often deemed less complicated, thus more likely to yield FSO. RCTs comparing ioECoG-tailored and non–ioECoG-tailored surgery are challenging for ethical considerations. Future methodologically sound observational research including large samples and controlling rigorously confounders may strengthen the evidence.

To address the challenge of simultaneous comparisons across multiple pathologies, we used an NMA approach that allowed us to integrate direct and indirect evidence. Consistency of the NMA was tested and observed in most direct and indirect comparisons. In line with our subanalysis at study level, the NMA showed ioECoG-tailored resection in case of tumors was superior to that of MCDs regarding achieving FSO. Similar results were found in a meta-analysis on temporal lobectomy, where the rate of achieving seizure freedom was 83% for tumor and 61% for MCD.^32^ A potential explanation might be that some subtypes of FCD, particularly FCD type 1, have been associated with poor prognosis.^5^ Overall, type 1 FCD is a more diffuse structural abnormality, and colocalization with functional cortex often leads to incomplete resection, which yields a higher chance of seizure recurrence.^33^ An IPDMA revealed that FCD-related epilepsy surgery with ioECoG tailoring was more likely to attain FSO than surgery without ioECoG.^24^ No such difference was found in tumor-related epilepsy surgery. These findings do not contradict our conclusions. Our study focused on ranking FSO rates of distinct pathology-related epilepsy after ioECoG-tailored surgery. Due to unavailability of data on distinct pathology of epilepsy without ioECoG-tailored surgery, we could not reproduce such IPDMA, and the added value of ioECoG in distinct pathologies was not explored in our study.

We demonstrated that complete resection of tissue generating IEDs on ioECoG was associated with FSO after epilepsy surgery. Previous studies suggested that the concomitant removal of lesion and adjacent epileptic tissue led to better seizure outcome compared with lesionectomy alone.^34,35^ In agreement with our result, a study showed that decrease in IEDs in postresection ioECoG was significantly associated with good outcome (Engel class I+II), especially in patients with temporal lesions.^36^ However, other conflicting study has not shown complete resection of spike-generating tissue could predict good seizure outcome.^37^ IEDs spread and can thus be difficult to delineate, and the resection itself may give rise to irritative spikes near the resection border.^15,16^ Some researchers therefore challenged IEDs as a reliable indicator of epileptogenic tissue.^38,39^ Interictal HFOs, particularly fast ripples, seem to be more specific for epileptogenicity in comparison with IEDs,^14,40^ and previous studies indicated that resection of areas with high rates of HFOs is associated with an increased chance of a good seizure outcome.^39,41,42^ HFOs are more difficult to visualize during surgery though, and thus, firm evidence is needed before clinical implementation. An RCT on ioECoG showed no superiority of HFOs compared with IEDs for the whole group, but after confounder correction for poor pathology prognosis, it did demonstrate noninferiority in the eTL subgroup.^29^ In addition, several studies showed that HFOs-on-spikes predicted postsurgical non–seizure freedom better than HFOs-not-on-spikes.^43^ The detection of HFOs may not necessarily reflect epileptogenic tissue only because HFOs in functionally eloquent areas may be physiologic.^44^ We should maybe refrain from comparing IEDs and HFOs regarding superiority and obtain the most valuable information for epilepsy surgery by integrating these and perhaps also other biomarkers.^45,46^

In contrast to previous studies and meta-analyses that convincingly demonstrated an increased odds ratio for seizure freedom in MRI-positive patients, we did not observe a significant difference in FSO rate between MRI-positive and MRI-negative epilepsy after ioECoG-tailored surgery.^31^ Limited statistical evidence supporting the existence of this difference may be attributed to the small sample size of the MRI-negative group (n = 57). Given MRI-positive lesions allows for better localization of epileptic tissue, such patients are considered good surgical candidates. This probably has led to a low number of surgeries in MRI-negative patients.^47^ In addition to this statistical aspect, we pose 2 explanations for this observation. One explanation might be incomplete resection of lesions situated within functional regions. In such cases, the strength of MRI positivity is limited. Another explanation might be that additional presurgical metabolic diagnostics (e.g., PET or SPECT) provided complementary information to the MRI.^48^ For example, a study by Feng et al. reported the ratios of obtaining Engel I were almost even (68.4% vs 68.3%) in the MRI-positive TL group and MRI-negative but PET-positive TL group.^49^ Thus, given those explanations, our observation, which was a nonsignificant trend in the expected direction, calls for caution in drawing conclusions about the equivalence of FSO after ioECoG-tailored surgery in MRI-positive and MRI-negative epilepsy.

There was no dichotomous meta-analytic evidence of a difference in FSO rate for TL vs eTL (Figure 3B), while study-level analysis (Table 1) indicated a trend toward a lower FSO rate in the eTL surgery group than in the TL group. One reason could be that the eTL surgery group involved only 3 studies with 89 patients. Another potential reason lies in the difference in statistical methodologies used. Specifically, the dichotomous meta-analysis adopted a fixed-effects model, whereas the meta-regression analysis applied a mixed-effects model, which accounts for the inherent interstudy heterogeneity. A possible bias in this comparison was that MTS accounted for the most with the TL surgery group, yet eTL epilepsy was mostly caused by tumor and cavernous malformations (CMs). The application of ioECoG for epilepsy surgery in case of MTS may not be as beneficial as for tumor and CMs (Table 1). A study by Cho et al. showed that 44.0% of patients with TL epilepsy surgery rendered seizure-free by ioECoG-tailoring, whereas 78.2% of the patients with eTL had seizure-free outcomes.^23^ Future investigations should strive to determine the effect of the anatomical location of epilepsy on the seizure outcome of ioECoG-tailored surgery, using adequately sized populations and taking into account other important variables such as pathology.

The primary strength of this meta-analysis lies in the inclusion of large number of studies and substantial patient sample size. We were able to use various statistical methods to investigate relations between predefined factors and effect size, examine sources of heterogeneity, and compare FSO in distinct pathologies by using NMA.

Our study has its limitations. First, the included studies could have suffered from selection bias in the choice for ioECoG. This inherent bias leads to a relatively broad conclusion without explicitly identifying specific patient populations for whom ioECoG could be the only suitable approach. The primary reason of this bias is the absence of an official guideline determining when ioECoG would be beneficial in guiding resection. Second, our study was largely composed of observational studies, limiting the scope of the available evidence. While an overall moderate-to-high quality of included studies according to Cochrane and NOS tool, the evidence of the estimates was rated at a low or very low certainty using GRADE. Third, we grouped studies using different classifications for FSO in meta-regression analysis, including different thresholds within the Engel classification, leading to heterogeneity (Table1). People experiencing auras or incidental seizures ended up in the FSO group for some studies and non–FSO group for other studies. The ambiguous reporting of seizure outcome (i.e., SFS) in 9 studies may have led to further inconsistencies in FSO. This may have affected the robustness of our results and biased our subgroup analyses. Fourth, studies with less than 10 patients and studies not differentiating between ioECoG and extraoperative ECoG were excluded, resulting in information loss. Fifth, we included studies with a minimal follow-up of 6 months. A follow-up of at least 1 year is preferred because this is presumed clinically relevant. The mean follow-up for all included studies was more than 1 year, but 11 studies contained some patients with shorter follow-up duration. We did not want to omit these studies based on few patients. Last, despite our efforts to quantify the source of variability through univariate meta-regression, we were unable to statistically analyze additional clinical variables that might be source of variability. Seizure frequency and duration of epilepsy, surgeon experience, use of antiepileptic medication, anesthetics, and electrode density of the used grids on seizure outcome were not thoroughly examined due to the limited frequency and inconsistent reporting. We advocate for further studies to explore these associations.

## Recommendations for Future Research

This meta-analysis may inspire using intraoperative ECoG more widely, including in tumor-associated epilepsy. Future research should further facilitate data analysis and synthesis. Retrospective and prospective cohort studies comparing ioECoG-tailored and non–ioECoG-tailored epilepsy surgery are needed to determine whether ioECoG truly adds value. Study data should reflect distinct pathology, MRI characteristics, anatomical locations of epilepsy surgery, and corresponding FSO for individual patients to help future meta-analysis on group comparisons. In addition, information on presurgically expected outcome and if and how ioECoG changed the original surgical plan is desirable. There is currently 1 RCT on ioECoG-tailored surgery, comparing HFOs with spikes. More trials or prospective cohort studies will be invaluable to establish the clinical benefit of epileptic ioECoG biomarkers. We encourage studies to identify patient populations that may not currently receive ioECoG but could potentially benefit from it.

## Appendix Authors

**Table:**

Name	Location	Contribution
Jiaojiao Guo, MD	Department of Neurology and Neurosurgery, University Medical Center Utrecht Brain Center, University Medical Center Utrecht, Part of ERN EpiCARE, the Netherlands	Drafting/revision of the article for content, including medical writing for content; major role in the acquisition of data; study concept or design; and analysis or interpretation of data
Ziyi Wang, MD	Department of Neurology and Neurosurgery, University Medical Center Utrecht Brain Center, University Medical Center Utrecht, Part of ERN EpiCARE, the Netherlands	Analysis or interpretation of data
Maryse A. van 't Klooster, PhD	Department of Neurology and Neurosurgery, University Medical Center Utrecht Brain Center, University Medical Center Utrecht, Part of ERN EpiCARE, the Netherlands	Drafting/revision of the article for content, including medical writing for content; study concept or design
Sandra M. Van Der Salm, MD, PhD	Department of Neurology and Neurosurgery, University Medical Center Utrecht Brain Center, University Medical Center Utrecht, Part of ERN EpiCARE, the Netherlands	Drafting/revision of the article for content, including medical writing for content
Frans S. Leijten, PhD	Department of Neurology and Neurosurgery, University Medical Center Utrecht Brain Center, University Medical Center Utrecht, Part of ERN EpiCARE, the Netherlands	Drafting/revision of the article for content, including medical writing for content
Kees P. Braun, MD, PhD	Department of Neurology and Neurosurgery, University Medical Center Utrecht Brain Center, University Medical Center Utrecht, Part of ERN EpiCARE, the Netherlands	Drafting/revision of the article for content, including medical writing for content
Maeike Zijlmans, MD	Department of Neurology and Neurosurgery, University Medical Center Utrecht Brain Center, University Medical Center Utrecht, Part of ERN EpiCARE; Stichting Epilepsie Instellingen Nederland (SEIN), the Netherlands	Drafting/revision of the article for content, including medical writing for content; study concept or design

## Glossary

CrI: credible interval
CM: cavernous malformations
eTL: extratemporal lobe
EEG: electroencephalography
FCD: focal cortical dysplasia
FSO: favorable seizure outcome
GRADE: Grading of Recommendations, Assessment, Development, and Evaluation
HFOs: high-frequency oscillations
ioECoG: intraoperative electrocorticography
IEDs: interictal epileptiform discharges
ILAE: International League Against Epilepsy
IPDMA: individual participant data meta-analysis
MCD: malformations of cortical development
MTS: mesiotemporal sclerosis
NOS: Newcastle-Ottawa Scale
NMA: network meta-analysis
OR: odds ratio
RCT: randomized controlled trial
SFS: seizure-free status
SOZ: seizure-onset zone
